# Collective Behaviours: Mediation Mechanisms Underlying the Influence of Descriptive and Injunctive Norms

**DOI:** 10.5334/irsp.806

**Published:** 2023-11-13

**Authors:** Lisa Selma Moussaoui, Katherine M. White, Olivier Desrichard

**Affiliations:** 1 Health Psychology Research Group, Faculty of Psychology and Education Sciences, University of Geneva, Switzerland; 2 School of Psychology & Counselling, Faculty of Health, Queensland University of Technology, Australia

**Keywords:** outcome expectancy, social influence, descriptive norm, injunctive norm, informational influence, normative influence

## Abstract

Conformity to descriptive and injunctive norms has been explained by informational and normative social influence. We argue that in addition to these two types of social influence, outcome expectancy can mediate descriptive norms’ impact on people’s intentions in the case of collective behaviours such as hand washing to prevent virus spread. Two studies manipulate norm type (descriptive vs injunctive) and norm level (low vs high) and show their effects on intention to perform the behaviour. In Study 1 (*N* = 216), outcome expectancy was positively influenced by descriptive norm and was associated with intention. In Study 2 (*N* = 731), outcome expectancy was influenced by descriptive but also by injunctive norm. Similar to Study 1, outcome expectancy was significantly associated with intention. Our data support the idea outcome expectancy is an important antecedent of intention and an additional mechanism underlying the effects of descriptive norms and, in some instances, injunctive norms.

Many of the world’s current challenges involve collective pro-social behaviours. Preventing the spread of epidemics is one example of contemporary societal problems that challenge our everyday behaviours and encourage us to change our lifestyles for the good of all. In this context, information on how the prescribed behaviour is perceived (injunctive norm) and adopted by others (descriptive norm) plays an important role (Howe et al., 2021). The effect of norms on individual behaviour is the focus of a long research tradition. However, knowledge about how these norms may act in the context of collective pro-social behaviours is limited, especially when it comes to understanding the underlying mechanisms. Yet, for scientists and practitioners, knowing how descriptive and injunctive norms influence collective pro-social behaviours is often critical. The present studies aimed to extend past research in the context of collective pro-social behaviours by investigating another potential mediator of conformity to the norm: outcome expectancy. We manipulated descriptive and injunctive norm levels in two studies and measured potential mediators. We expected that classical mediators of normative influence (e.g., informational social influence and normative social influence) would operate in both descriptive and injunctive norm manipulations and that outcome expectancy would mediate the effect of descriptive norm only.

## Social Influence and Social Norms

Social influence has been studied widely since Sherif’s (1935) and Asch’s (1951) classical studies. Normative social influence is defined as conforming to the expectations of others, while informational social influence is the process of relying on others to obtain information about reality (Deutsch & Gerard, 1955). Cialdini, Reno, and Kallgren (1990), in turn, distinguished two types of norms: descriptive versus injunctive. Descriptive norm is based on what is done by others. For example, in the COVID-19 context, the frequent mention of vaccine coverage in the news (i.e., the percentage of vaccinated people in the region or the country) represented information reflecting descriptive norms. Injunctive norm is what ought to be done. In the same context, injunctive norm could be the perception one has about how many others are favourable towards the vaccine. The authors associated descriptive norm with informational social influence, while injunctive norm was associated with normative social influence.

For descriptive norm, it has been proposed that if everybody except one person holds the same opinion, it is legitimate to infer the majority is correct (under the condition there is no plausible reason to suspect they would not tell the truth). This reasoning was described by Asch’s (1956) participants’ comments post-experiment (‘they felt the majority had to be right and they had to be wrong’, Cialdini & Trost, 1998:163). Social Comparison Theory by Festinger (1954) also predicts that to evaluate one’s behaviour correctness; one might compare it to others’ actions that constitute the standard. Thus, several lines of research converge to state that descriptive norms influence behaviour through informational social influence, with people pursuing the goal of effective action and accurate decision-making (Cialdini & Trost, 1998).

Injunctive norms are described by Cialdini et al. (1990) as driving behaviour with social rewards and sanctions. Injunctive norm states (explicitly or implicitly) what is prescribed and what is proscribed. The primary mechanism for conformity to injunctive norms is normative social influence (Cialdini et al., 1990). Thus, people would follow injunctive norms to build and maintain social relationships (Cialdini & Trost, 1998). Later work has opened avenues for crossed mechanisms, such that descriptive norms might also be followed for normative social influence motivation (Göckeritz et al., 2010).

Different conceptualisations of these processes have arisen, such as self-categorisation theory. The two types of influence are conceptualised as one process, referent informational influence related to group membership and social identity, instead of being distinct processes (Hogg & Turner, 1987; Turner, 1985). Despite other lines of inquiry, much social influence theorising still rests on the seminal concepts of normative and informational social influence as two processes.

## Existing Work on Mediators of Social Norm’s Effect

The influence of norms on behaviour has mainly been conceptualised as unconscious. For example, in a study by Nolan et al. (2008), participants rated others’ behaviour as the least important reason to save energy, while the results showed that perceived descriptive norm was the strongest determinant of energy savings. However, some lines of research imply deliberate processing of normative information (Schultz et al., 2008). Despite the extensive theoretical elaboration on norm conformity, few studies have explicitly tested mediation mechanisms underlying injunctive and descriptive norms effects (for exceptions, see Culiberg & Elgaaied-Gambier, 2016; Doran & Larsen, 2015; Howe et al., 2021; Lee & Paek, 2014). Schultz et al. (2008) described the research on the processes underlying normative influence as sparse (p. 402).

Rimal (2008) proposed that outcome expectations mediate the relationship between descriptive norms and intention to act (in this case, alcohol drinking). The hypothesised mechanism is that seeing others performing the behaviour and having desirable outcomes would lead the person to engage in the behaviour from fear of being deprived of those benefits. Indeed, the results showed outcome expectations (regarding perceived benefits and anticipatory socialisation) partially mediated the relationship between descriptive norms and intention to drink alcohol. Huber et al. (2022) similarly showed outcome expectations (both positive and negative) about the nonmedical use of prescription drugs mediates the effect of social norms. Of note is that the behaviours in these two studies were individual (alcohol consumption and drug use). Few studies have examined the potential mediators between norms and behaviours for collective, pro-social behaviours. Below, we argue that outcome expectancy is especially relevant in the case of collective behaviours.

## Norm Effects on Collective Pro-Social Behaviours

Several studies have examined the effects of descriptive and injunctive norms on behaviours that could be classified as collective and pro-social such as conservation behaviour (for a recent meta-analysis, see Niemiec et al., 2020); blood donation (Reingen, 1982; Von Borgstede et al., 1999); and donating money to charity (Croson et al., 2009; Howe et al., 2021; Reingen, 1982) or contribute to a monetary public good (Croson & Shang, 2013; Von Borgstede et al., 1999). Collective behaviours can be illustrated by a quote from Koletsou and Mancy (2011): ‘Climate change, like many large-scale problems, induces a situation in which individuals have only a small influence and goals can only be achieved through collective action.’ (p.191).

Howe et al. (2021) showed that when a normative appeal is framed as collective effort (i.e., mentioning the percentage of others donating money for a cause and sentences such as ‘let’s do it together, join in’), the effect on behaviour is stronger than a standard normative appeal (only mentioning the percentage of others donating) or a control condition (no mention of others behaviour). The authors also show that this effect is mediated through the feeling of working together. Specifically for large-scale social dilemmas, Von Borgstede et al. (1999) observed that the more others cooperate, the more one is willing to cooperate too. They explained this result with the idea that when many people cooperate, individual action will probably be a valuable contribution to the collective outcome, while if only a few people participate in the common good, individual efforts may be useless. Others have also proffered this notion (Burn, 1991; Hanss et al., 2016; Staats et al., 1996), but it was never experimentally tested except by Moussaoui and Desrichard (2017) and Soliman et al. (2018). Moussaoui and Desrichard showed in two studies the effect of descriptive norms on environment-friendly behaviour is mediated by outcome expectancy: the more people cooperate on a collective goal, the higher the expectations that individual effort will be useful. Soliman et al. tested how social norms could reduce ‘drop in the bucket’ perception toward action to mitigate climate change, with consequences distant in time. They found the drop in the bucket perception was slightly reduced in the condition mentioning that 85% of Canadians have begun to behave more sustainably. As only two papers explored the idea of outcome expectancy being influenced by descriptive norm, and only in the domain of pro-environmental behaviours, we decided to replicate and extend those results. The present paper goes beyond the existing work because we include injunctive norms, with the goal of testing that the effect is specific to descriptive norm as our theoretical reasoning implies. Is the effect specific to the mention of how many others are cooperating or is it that any mention of norms such as the proportion of others’ approval (i.e., injunctive norms) can also be mediated by outcome expectancy? To the best of our knowledge, the literature does not provide an answer to this question. In terms of replication, following Hüffmeier et al. (2016) typology, we propose a constructive replication of Moussaoui and Desrichard’s (2017) study. Aside from the fact that more evidence contributes to the robustness of the effect, our study aims to determine if the descriptive aspect of norm is necessary or if injunctive norms have the same effect. In addition, testing the effect on a different behaviour will allow generalisation to other collective pro-social behaviours.

## The Present Study

**Figure 1:**
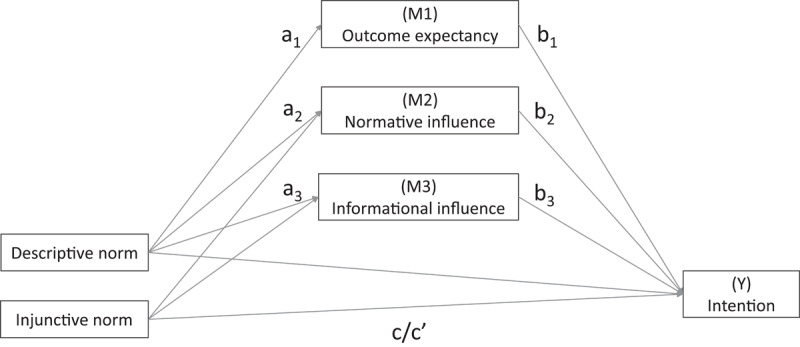
The Parallel Mediation Model Hypothesised.

This paper investigates the mediating mechanisms responsible for injunctive and descriptive norms’ effects on collective pro-social behaviours. The hypothesis is that descriptive norms are mediated by outcome expectancy in addition to informational and normative influence. In contrast, injunctive norm is expected to be mediated only by informational and normative influence (not outcome expectancy). This expectation is because knowing how many people approve of a behaviour (i.e., injunctive norm) tells you little about the probability the collective goal will be reached. On the contrary, knowing how many people are doing a behaviour (i.e., descriptive norm) is an indicator of the probability or likelihood the collective goal will be reached. The hypothesised model is presented in Figure 1.

We test these hypotheses on hand-washing behaviour to prevent virus spread. Hand-washing and other protective behaviours in the context of the COVID-19 pandemic have been presented as part of large-scale collective action or social dilemmas (Harring et al., 2021; Moussaoui et al., 2020). Collective action problems or social dilemmas imply individual interests are not the same as the collective best long-term interests. From a personal perspective, it is a hassle to wash my hands every time I sneeze; thus, I might be incentivised not to do it. However, regarding societal interest, hand-washing prevents the spread of viruses and bacteria (Aiello et al., 2010; Hugonnet & Pittet, 2000; Tabish & Basch, 2020; Xun et al., 2021). Moreover, as it is a repeated action that has to be also performed by other people (e.g., because many people touch a door handle or handholds in public transport), it seems an appropriate behaviour to test our hypothesis.

Two studies testing this hypothesis are presented. Those two studies result from a line of studies available online^1^ for transparency.

## Study 1

### Method

The study received approval from the University ethics committee (n°1800000207), and the study’s hypothesis and analysis plan were preregistered on Aspredicted https://aspredicted.org/blind.php?x=ch595g.^2^

#### Participants

Using Schoemann et al. (2017) method, we calculated the sample size required to test our model. Fixing power at .80 and correlations between variables at .2, a value considered small-to-moderate, we obtained a required *N* of 365^3^ (details of the calculation are provided in the Appendix). Students of an Australian University were recruited in exchange for a prize draw or course credit. A total of 342 students started the survey, but 44 did not complete it and, thus, did not provide their consent for the use of their data after the final debriefing. In addition, 10 participants were excluded based on the attention check (an item explicitly asking not to click on an answer to detect participants not reading the items), and 69 failed the manipulation check. Three participants were further excluded based on outlier detection. The final sample size is *N =* 216.^4^ Because our sample size is smaller than the one required, we conducted a sensitivity power analysis to estimate the achieved power. Considering the correlations between our variables and standard deviations, for path a1b1, power = .64; path a2b2, power = .86; and for path a3b3, power = .07 (details provided in the Appendix).

#### Procedure

The study was conducted online. Participants read the information and consent form before starting the study. To prevent any demand characteristics, we advertised the study as being about ‘how people understand and use graphical information to make decisions’. After consenting to participate, background information was measured (age, gender, current country of residency). Then participants were exposed to information about descriptive norm (between-participant variable, two levels: low (20–30%) or high (60–70%), and injunctive norm (between-participant variable, two levels: low (20–30%) or high (60–70%). Each participant saw a percentage for each type of norm; thus, type of norm (descriptive and injunctive) was a within-participant variable. They then had to answer manipulation checks to ascertain they correctly understood the information presented on the graphs. Participants answered items measuring their perceived outcome expectancy (i.e., the extent to which they think their behaviour will impact the goal), informational and social normative influences items, and their intention to enact the target behaviour. We also inserted an item to check if participants were reading the survey items properly (asking the participant explicitly NOT to answer anything for the item, with participants clicking on any answer for this item being excluded). After the study was over, participants were debriefed on the real goal of the study and had the opportunity to confirm their agreement with the use of their data. Accurate descriptive and injunctive norm information statistics were finally provided to prevent participants from leaving the experiment with false statistical data in mind.

#### Materials

**Norm manipulation**. The following instructions were provided to participants: *Results of a recent survey conducted in Australia among a representative sample of the general population will be presented to you on the next pages.[…]. To make the survey as short as possible, you will be randomly allocated to answer to opinion questions about only one of the three behaviours*. The last sentence refers to non-target behaviours (condom use and skin cancer screening) added as part of the cover story (Smith & Louis, 2008; White et al., 2002).

Participants saw graphical information about the target behaviour (hand-washing) and non-target behaviours, with information about others’ behaviours and opinions. This information was presented as actual survey results while, in fact, this was the experimental manipulation of our independent variable. The computer randomised participants into one of four conditions, with graphical presentation of varying levels of norms (low injunctive norm level & low descriptive norm level; low injunctive norm level & high descriptive norm level; high injunctive norm level & low descriptive norm level; high injunctive norm level & high descriptive norm level). In addition to graphical information, participants also read illustrative quotes from people who purportedly completed the survey that produced the graphical information. The quotes expressed the same level of norms as the graphs. The quotes were expected to help strengthen the normative manipulation (based on Smith & Louis, 2008; White et al., 2002).

Answers for the manipulation check items (before exclusion of participants failing the check) show the induction worked as the means significantly differ between groups. Participants exposed to high descriptive norm information had higher means than those exposed to low descriptive norm (*F*(1, 285) = 7.31, *p* = .008). Similarly, the injunctive norm induction influenced answers on the check: participants in the high injunctive norm condition had higher answers on the manipulation check than participants in the low injunctive norm condition (*F*(1, 285) = 13.76, *p* < .001). The means for each experimental condition are presented in Table 1.

**Table 1:** Manipulation check values according to experimental conditions (Study 1).


MANIPULATION CHECK MEASURE	EXPERIMENTAL CONDITION			

	HIGH DESCRIPTIVE, HIGH INJUNCTIVE*M (SD)*	HIGH DESCRIPTIVE, LOW INJUNCTIVE*M (SD)*	LOW DESCRIPTIVE, HIGH INJUNCTIVE*M (SD)*	LOW DESCRIPTIVE, LOW INJUNCTIVE*M (SD)*

Descriptive norm	7.00 (0.33)	6.97 (0.29)	4.15 (0.94)	3.49 (1.40)

Injunctive norm	7.88 (0.37)	3.86 (1.58)	7.81 (0.79)	4.46 (1.38)


*Note*. Scale coding: 1 = 1–9%, 2 = 10–19%, 3 = 20–29%, 4 = 30–39%, 5 = 40–49%, 6 = 50–59%, 7 = 60–69%, 8 = 70–79%, 9 = 80–89%, 10 = 90–99%.

**Outcome expectancy**. Three items measured the perception of outcome expectancy toward hand-washing. For example, ‘Given that [31.7% / 70.3%] of people are in favour of people frequently washing their hands or using sanitiser after coughing or sneezing and that [23.1% / 67.1%] of people frequently wash their hands/use sanitiser after coughing or sneezing, if you washed yours/used sanitiser, how useful do you think your action would be in order to contribute to the goal of preventing the spread of contagious viruses in Australia?’, seven-point answer scale ranging from *extremely useless* (1) to *extremely useful* (7). The reliability of these items was good (α = .858).

**Normative influence**. Three items measured the perception of normative influence, e.g., ‘Given that [31.7% / 70.3%] of people are in favour of people frequently washing their hands or using sanitiser after coughing or sneezing and that [23.1% / 67.1%] of people frequently wash their hands/use sanitiser after coughing or sneezing, I think others would disapprove of me if I did NOT wash my hands/use sanitiser after coughing or sneezing’, scale ranging from *strongly disagree* (1) to *strongly agree* (7). The reliability of these items was good (α = .833).

**Informational influence**. Three items measured the perception of informational influence; for example, ‘Given that [31.7% / 70.3%] of people are in favour of people frequently washing their hands or using sanitiser after coughing or sneezing and that [23.1% / 67.1%] of people frequently wash their hands/use sanitiser after coughing or sneezing, NOT washing hands/using sanitiser after coughing or sneezing is a mistake’, scale ranging from *strongly disagree* (1) to *strongly agree* (7). The reliability of these items was satisfactory (α = .740).

**Intention**. Three items measured intention to perform the target behaviour in order to contribute to a collective goal (i.e., preventing the spread of a contagious virus); for example, ‘Given that [31.7% / 70.3%] of people are in favour of people frequently washing their hands or using sanitiser after coughing or sneezing and that [23.1% / 67.1%] of people frequently wash their hands/use sanitiser after coughing or sneezing, it is extremely likely that I would wash my hands/use sanitiser after coughing or sneezing in order to contribute to the goal of preventing the spread of contagious viruses in Australia’, scale ranging from *strongly disagree* (1) to *strongly agree* (7). The reliability of these items was good (α = .921).

### Results

Following recommendations by Yzerbyt et al. (2018) for testing mediational models, we first report results from the component approach (i.e., individual parameter estimates), and then results obtained via the index approach (i.e., single mediational index, using the PROCESS macro). Step 1 aims to test if the component paths of the indirect effect are significant. If so, Step 2 allows examining the magnitude of the indirect effect.

Contrary to what was preregistered, we did not control for past behaviour in the main analysis for Study 1. Due to an error, the measure of past behaviour was placed at the end of the survey. Because past behaviour items were placed after the experimental manipulation, our independant variables (IVs) could have influenced participants’ reports of their past behaviours. We tested this suspicion and found that, indeed, the main effect of descriptive norms on past behaviour was not null, *F*(1,210) = 3.15, *p* = .077, ƞ*^2^_p_* = .015, as well as the interaction between injunctive and descriptive norms, *F*(1,210) = 3.12, *p* = .079, ƞ*^2^_p_* = .015. Thus, it seemed that controlling for past behaviour could have reduced the experimental manipulation effect on the dependant variable (DV) (similar to the decrease in the association between the IV and DV when adding a mediator in a regression). To provide the full picture, we conducted a sensitivity analysis to examine the impact of controlling for past behaviour and present the results in the Appendix.

Descriptives values for our dependent variable and the mediators are presented in Table 2.

**Table 2:** Means, standard deviation, and correlations with confidence intervals (Study 1).


VARIABLE	*M*	*SD*	1	2	3

**1.** Outcome expectancy	4.32	1.30			

**2.** Normative influence	4.93	1.20	.281** [.15, .40]		

**3.** Informational influence	5.13	1.05	.283** [.15, .40]	.459** [.35, .56]	

**4.** Intention	5.25	1.27	.380** [.26, .49]	.554** [.45, 64]	.651** [.57, .72]


*Note:* ** < .001.

#### Component approach results

Results are presented in Table 3.

**Table 3:** Component approach results (Study 1).


VARIABLES	OUTCOME EXPECTANCY			NORMATIVE INFLUENCE			INFORMATIONAL INFLUENCE			INTENTION		
			
	*F*	*p*	ƞ*2p*	*F*	*p*	ƞ*2p*	*F*	*p*	ƞ*2p*	*F*	*p*	ƞ*2p*

Step 1 df*s* (1, 210)												

Descriptive norm	6.92	.009	.001	7.75	.006	.036	0.35	.554	.002	0.75	.387	.004

Injunctive norm	2.35	.127	.011	11.97	.001	.054	1.78	.183	.008	0.12	.728	.001

Interaction	0.20	.657	.001	4.43	.037	.021	6.77	.010	.031	4.55	0.34	.021

Gender	2.14	.145	.010	.001	.975	.000	0.37	.544	.022	1.18	.278	.006

Age	14.83	<.001	.066	5.53	.020	.026	5.19	.024	.024	1.39	.240	.006

Step 2 df*s* (1, 212)												

Outcome expectancy										29.54	<.001	.122

Normative influence										89.06	<.001	.296

Informational influence										147.56	<.001	.410

Step 3 df*s* (1, 207)												

Descriptive norm										0.04	.836	.000

Injunctive norm										4.80	.030	.023

Interaction										0.09	.764	.000

Outcome expectancy										10.78	.001	.050

Normative influence										32.35	<.001	.135

Informational influence										70.71	<.001	.255


***Path a_1_: effect of descriptive and injunctive norms on outcome expectancy.*** Testing the effect of X on M1, we expected a positive main effect of descriptive norm level on outcome expectancies and simple effects of descriptive norm at each level of injunctive norm (i.e., no interaction). Injunctive norm was not expected to produce differences in outcome expectancy. As expected, injunctive norm level did not have a significant effect, while the higher the descriptive norm, the higher the outcome expectancies. The interaction between injunctive and descriptive norm was non-significant.

***Path a_2_: effect of descriptive and injunctive norms on normative influence.*** We predicted positive main effects of each type of norm, as well as simple effects on normative social influence. As expected, normative social influence was influenced by injunctive norm level, as well as by descriptive norm level. The interaction between both norm types was significant. Analysis of simple effects showed normative influence scores of participants in the low injunctive norm condition did not differ when they were in the high descriptive norm condition compared to those in the low descriptive norm condition (difference in means: 0.11, *p* = .640). Participants in the high injunctive norm condition had 0.76 higher normative influence scores in the high descriptive norm condition than in the low descriptive norm condition (*p* < .001). Conversely, participants in the low descriptive norm condition had equivalent normative influence scores when they were in the high injunctive norm condition compared to those in the low injunctive norm condition (difference in means: 0.21, *p* = .333). Participants in the high descriptive norm condition had 0.86 higher normative influence scores in the high injunctive norm condition than those in the low injunctive norm condition (*p* < .001).

***Path a_3_: effect of descriptive and injunctive norms on informational influence.*** The results for informational social influence showed this variable was unexpectedly not influenced by injunctive norm level, nor by descriptive norm level. However, the interaction between both norm types was significant. Analysis of simple effects showed that participants in the low injunctive norm condition had 0.45 lower informational influence scores in the high descriptive norm condition than those in the low descriptive norm condition (*p* = .033), an effect in the opposite direction to what was predicted. Participants in the high injunctive norm condition had similar informational influence scores in the high descriptive norm condition compared to participants in the low descriptive norm condition (difference in means: 0.28, *p* = .137). Participants in the low descriptive norm condition had equivalent informational influence scores when in the high injunctive norm condition compared to those in the low injunctive norm condition (difference in means: -.17, *p* = .384). Participants in the high descriptive norm condition had 0.56 higher informational influence scores in the high injunctive norm condition than those in the low injunctive norm condition (*p* = .006).

***Path b_1_: effect of outcome expectancy on intention.*** Testing the effect of M1 on Y, we expected a positive association between outcome expectancy and intention. Results supported this hypothesis as the effect was statistically significant.

***Path b_2_: effect of normative influence on intention.*** Similarly, we expected a positive association between normative social influence (M2) and intention, and this effect was statistically significant.

***Path b_3_: effect of informational influence on intention.*** The path from the third mediator, informational social influence, to intention was also significant and positive.

***Path c: effect of descriptive and injunctive norm on intention***. Regarding the effect of norms on intention, we expected that only when both injunctive norm and descriptive norm are high should intention increase. No hypotheses were made regarding possible main effects. The ANOVA on intention showed no significant main effects for descriptive norm and injunctive norm. The interaction was significant. Means by group are: *M_lowInjlowDescr_* = 5.33 (*SD* = 0.18); *M_lowInjhighDescr_* = 5.12 (*SD* = 0.18); *M_highInjlowDescr_* = 5.03 (*SD* = 0.17); *M_highInjhighDescr_* = 5.54 (*SD* = 0.16). Planned contrast showed that the mean in the high injunctive high descriptive norms condition was significantly different from the three other groups’ means ((–1 –1 –1 3) *t =* 2.03, *p* = .044), while the residual contrasts were not significant (respectively, (–1 –1 2 0) *t =* –0.29, *p* = .773; (–1 1 0 0) *t =* –1.24, *p* = .216).

***Path c’: effect of descriptive and injunctive norm on intention with mediators controlled for***. Finally, we tested the effect of the mediators on intention after controlling for the level of descriptive norm, level of injunctive norm, and their interaction. The data revealed a main effect of outcome expectancy when entered as a predictor of intention, as well as main effects of normative and informational social influence. The main effect of descriptive norm was non-significant, while injunctive norm main effect became significant. The interaction between the two norm types was not significant.

#### Index approach results

**Figure 2:**
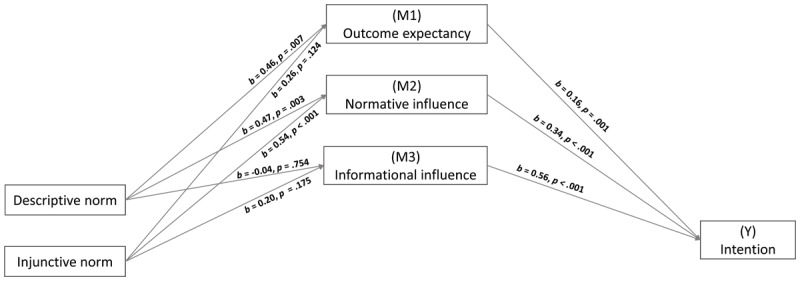
Parallel Mediation Model PROCESS Output (Study 1).

We predicted the descriptive norm effect would be mediated by normative social influence, informational social influence, and outcome expectancy. Meanwhile, normative and informational social influences only (not outcome expectancy) would mediate the effect of injunctive norms on intention. A PROCESS (v.3.3) macro was performed to analyse the parallel mediation. Coefficients are presented in Figure 2.

Indirect effects show outcome expectancy mediated the link between descriptive norm level and intention, *b* = 0.089, 95% CI [0.01 to 0.16]. Normative social influence also mediated the link between descriptive norm level and intention, *b* = 0.16, 95% CI [0.04 to 0.30], while this was not the case for informational social influence, *b* = –0.03, 95% CI [–0.18 to 0.13]. The direct effect of descriptive norm on intention was non-significant when mediators were entered in the model, *b* = –0.02, 95% CI [–0.26 to 0.22], *p* = .857.

Indirect effects show outcome expectancy and informational social influence did not mediate the link between injunctive norm level and intention, respectively *b* = 0.04, 95% CI [–0.01 to 0.12] and *b* = 0.11, 95% CI [–0.04 to 0.28], but that normative social influence did, *b* = 0.19, 95% CI [0.07 to 0.33]. The direct effect of injunctive norm on intention was significant when mediators were entered in the model, *b* = -0.27, 95% CI [–0.52 to –0.03], *p* = .029.

### Discussion

Study 1 showed that intention to wash hands was the highest when both injunctive and descriptive norms were presented as high. The effect of descriptive and injunctive norms on outcome expectancy conformed to our predictions: only descriptive norms level influenced outcome expectancy, not injunctive norm level. As expected, when outcome expectancy was entered as a predictor, it significantly impacted intention.

In accordance with the literature (e.g., Cialdini et al., 1990; Göckeritz et al., 2010), normative social influence was influenced by descriptive and injunctive norms, but this was surprisingly not the case for informational social influence. It is possible in our study the combination of two normative information sources played a role in how people perceived the behaviour as the right thing to do. In the literature, informational social influence has mostly been studied as predicted by descriptive norm only, contrary to normative social influence, which, although in the initial theoretical conception is supposed to be influenced exclusively by injunctive norm, has also been shown to be influenced by descriptive norm (Göckeritz et al., 2010). It is possible the two norms combination cancelled the effect of descriptive norms on informational social influence.

It is important to note Study 1 was underpowered relative to our calculations (N = 216 instead of N = 365); thus, the results should be interpreted cautiously and in a cumulative logic with future studies’ results. In particular, the lack of significant effect of injunctive norm on outcome expectancy, which we hypothesised, needs to be replicated in a study with stronger statistical power.

Past behaviour had a significant effect on all dependent variables, notwithstanding the results of the analysis were very similar when this variable was entered as control (results presented in the sensitivity analysis). Because past behaviour is theoretically a strong predictor of behaviour (Ouellette & Wood, 1998), we considered it important to move the past behaviour item at the beginning of the survey for the next study to prevent the influence of our experimental manipulation on this control variable.

## Study 2

### Method

We replicated the first study with a bigger sample size to have better statistical power as in the first study. We also moved past behaviour item to the very beginning of the survey before the IVs manipulation to prevent the issue that it would be influenced by the IVs and interfere in the analyses. Otherwise, all the material remained the same as in Study 1. Study 2 was preregistered at https://aspredicted.org/TZY_16G.

#### Participants

Participants were recruited on the crowdsourcing platform Prolific (Palan & Schitter, 2018). Participants had to be located in Australia to be able to take part, and they received two AUD in exchange for their participation.

We conducted a power analysis (Schoemann et al., 2017), setting power at .95 and using the same correlation values as in the a-priori analysis for Study 1. This analysis resulted in recommending at least 540^5^ participants (details in the Appendix). Because we had an exclusion rate of 26% in Study 1, we aimed for a sample size of at least 680 participants.

On Prolific, when we launched the Study, 1,253 participants matched the criteria (being located in Australia, and not having taken part in a similar study conducted by our team).^6^ We recruited 863 participants during the planned recruitment phase (August 31 to October 31). As preregistered, we stopped the data collection at the planned end date and proceeded with the analysis, given we had recruited more than 50% of the expected sample. Exclusions based on the check items in Study 2 (14%) were less than the 26% rate expected based on Study 1. A sensitivity power analysis was conducted, showing we had a power of 1 for path a1b1; .78 for path a2b2; and .19 for path a3b3 with our sample size of 731.

### Results

Similarly to Study 1, the induction of descriptive and injunctive norms influenced participants’ answers on the manipulation check: for descriptive norm (*F*(1, 854) = 13.46, *p* < .001) and injunctive norm (*F*(1, 854) = 31.50, *p* < .001) (see Table 4).

**Table 4:** Manipulation check values according to experimental conditions (Study 2).


MANIPULATION CHECK MEASURE	EXPERIMENTAL CONDITION			

	HIGH DESCRIPTIVE, HIGH INJUNCTIVE *M (SD)*	HIGH DESCRIPTIVE, LOW INJUNCTIVE *M (SD)*	LOW DESCRIPTIVE, HIGH INJUNCTIVE *M (SD)*	LOW DESCRIPTIVE, LOW INJUNCTIVE *M (SD)*

Descriptive norm	6.99 (0.34)	6.96 (0.40)	4.14 (0.69)	3.33 (1.10)

Injunctive norm	7.97 (0.22)	3.55 (1.41)	7.94 (0.34)	4.29 (0.95)


*Note*. Scale coding: 1 = 1–9%, 2 = 10–19%, 3 = 20–29%, 4 = 30–39%, 5 = 40–49%, 6 = 50–59%, 7 = 60–69%, 8 = 70–79%, 9 = 80–89%, 10 = 90–99%.

Descriptives values for our dependent variable and the mediators are presented in Table 5.

**Table 5:** Means, standard deviation, and correlations with confidence intervals (Study 2).


VARIABLE	*M*	*SD*	1	2	3

**1.** Outcome expectancy	4.54	1.40			

**2.** Normative influence	4.80	1.34	.332** [.27, .40]		

**3.** Informational influence	5.23	1.14	.425** [.36, .48]	.491** [.43, .54]	

**4.** Intention	5.25	1.43	.498** [.44, .55]	.418** [.36, .48]	.649** [.61, .69]


*Note*. ** < .001.

#### Component approach results

Statistical values are presented in Table 6.

**Table 6:** Component approach results (Study 2).


VARIABLES	OUTCOME EXPECTANCY			NORMATIVE INFLUENCE			INFORMATIONAL INFLUENCE			INTENTION		
			
	*F*	*p*	ƞ*2p*	*F*	*p*	ƞ*2p*	*F*	*p*	ƞ*2p*	*F*	*p*	ƞ*2p*

Step 1 df*s* (1, 724)												

Descriptive norm	38.91	<.001	.051	40.70	<.001	.053	2.08	.150	.003	19.24	<.001	.026

Injunctive norm	23.88	<.001	.032	138.23	<.001	.160	23.30	<.001	.031	9.80	.002	.013

Interaction	0.15	.694	.000	1.64	.201	.002	0.03	.866	.000	2.27	.132	.003

Gender	14.36	<.001	.019	4.02	.045	.006	4.88	.027	.007	13.04	<.001	.018

Age	13.16	<.001	.018	0.00	.971	.000	0.63	.428	.001	0.23	.632	.000

Past behaviour	55.50	<.001	.071	28.16	<.001	.037	159.07	<.001	.180	563.85	<.001	.438

Step 2 df*s* (1, 726)												

Outcome expectancy										169.50	<.001	.189

Normative influence										119.81	<.001	.142

Informational influence										283.45	<.001	.281

Step 3 df*s* (1, 721)												

Descriptive norm										2.86	.091	.004

Injunctive norm										2.74	.098	.004

Interaction										3.45	.064	.005

Outcome expectancy										62.23	<.001	.079

Normative influence										14.90	<.001	.020

Informational influence										124.30	<.001	.147


***Path a_1_: effect of descriptive and injunctive norms on outcome expectancy.*** Results supported our prediction that descriptive norm would have a significant positive effect on outcome expectancy. However, we hypothesised that injunctive norm should not affect outcome expectancy, while results show a positive effect on outcome expectancy. The interaction effect was non-significant.

***Path a_2_: Effect of descriptive and injunctive norms on normative influence.*** As expected, normative social influence was influenced by injunctive norm level and by descriptive norm level. The interaction was not significant.

***Path a_3_: Effect of descriptive and injunctive norms on informational influence.*** The pattern is different for informative social influence: injunctive norm’s main effect is significant, but not descriptive norm’s main effect. The interaction is not significant.

***Path b_1_: Effect of outcome expectancy on intention.*** Testing the effect of the first mediator on Y, we found a positive and significant association between outcome expectancy and intention.

***Path b_2_: Effect of normative influence on intention.*** Similarly, we expected a positive association between normative social influence, the second mediator, and intention. This effect was statistically significant.

***Path b_3_: Effect of informational influence on intention.*** Informational social influence, the third mediator, was also significant and positively associated with intention.

***Path c: Effect of descriptive and injunctive norm on intention.*** Intention was positively predicted by the level of each type of norm. We predicted that intention should be the highest when both descriptive and injunctive norms are high, but the interaction effect was not significant. Means’ pattern suggests that, instead of interaction, the effect of the two types of norm is additive: *M_lowInjlowDescr_* = 5.01 (*SD* = 0.08); *M_lowInjhighDescr_* = 5.23 (*SD* = 0.08); *M_highInjlowDescr_* = 5.14 (*SD* = 0.08); *M_highInjhighDescr_* = 5.59 (*SD* = 0.08).

***Path c’: Effect of descriptive and injunctive norm on intention with mediators controlled for.*** When adding the mediators as predictors in addition to injunctive and descriptive norms, each mediator was a significant predictor of intention. When mediators were included in the model, norms’ main effects and interaction were not significant.

#### Index approach results

**Figure 3:**
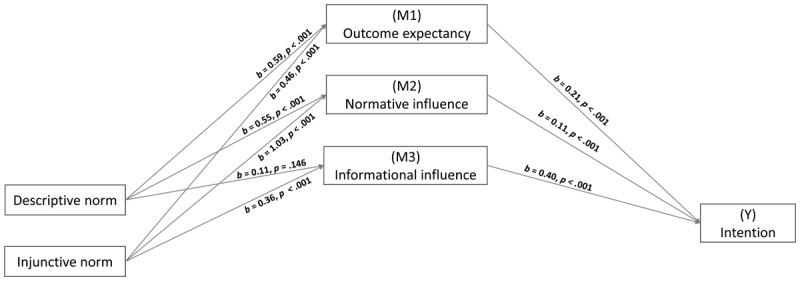
Parallel Mediation Model PROCESS Output (Study 2).

Coefficients are presented in Figure 3. All pathways conform to the hypothesis, except the following two: the significant effect of injunctive norm on outcome expectancy (which was expected to be non-significant) and the effect of descriptive norm on informational influence, which is not significant and was predicted to be significant.

Indirect effects show outcome expectancy mediated the link between descriptive norm level and intention, *b* = 0.12, 95% CI [0.08 to 0.18]. Normative social influence also mediated the link between descriptive norm level and intention, *b* = 0.06, 95% CI [0.02 to 0.11], while this was not the case for informational social influence, *b* = 0.04, 95% CI [–0.02 to 0.11]. The direct effect of descriptive norm on intention was non-significant when mediators were entered in the model, *b* = 0.12, 95% CI [–0.01 to 0.25], *p* = .070.

Indirect effects show that, contrary to our hypothesis, outcome expectancy mediates the link between injunctive norm level and intention, *b* = 0.10, 95% CI [0.05 to 0.15]. As expected, normative and informational social influence mediated the effect of injunctive norm level on intention, respectively *b* = 0.11, 95% CI [0.04 to 0.19], and *b* = 0.14, 95% CI [0.08 to 0.21]. The direct effect of injunctive norm on intention was non-significant when mediators were entered in the model, *b* = -0.11, 95% CI [–0.25 to 0.02], *p* = .102.

### Discussion

The second study was conducted to replicate with a bigger sample the results obtained in Study 1. Results of the second study confirmed the effect of descriptive norm is mediated through outcome expectancy as hypothesised. However, contrary to the hypothesis and Study 1 results, the effect of injunctive norm is mediated through outcome expectancy in Study 2. Similar to the first study, normative influence was predicted by both types of norms, while informational influence was only predicted by injunctive norms. This last effect does not fit with the theoretical background, which states that descriptive norm predicts informational influence. Nevertheless, as expected, both informational and normative influence predicted intention.

Study 2 has some limitations. First of all, despite an increased power compared to Study 1, we did not reach the planned sample size because there were too few available participants from a specific country on the platform where we recruited. However, a sensitivity analysis suggests we have enough power to detect small effect sizes. Second, it is important to note that Study 1 was conducted before the SARS-COV2 crisis, while Study 2 was conducted two years after the pandemic’s beginning. This time lag has several implications. The context might first induce a global increase in the intention to wash hands and the perceived level of adherence and approval by others. The hand hygiene items might not have resonated similarly in Studies 1 and 2 because it has become a protective behaviour widely advocated during the pandemic. Secondly, as discussed above, it can also have produced an effect where the distinction between injunctive and descriptive norms is blurred and explains some differences between the studies.

## General Discussion

Two studies investigated the mechanisms underlying the effects of descriptive and injunctive norms on people’s intentions to perform a collective behaviour, hand-washing to prevent the spread of a disease. To assist in explaining the processes underlying the effect of norms on intentions, we tested the hypothesis that beliefs of how much impact the individual action has on the collective goal partially explain the effect of descriptive norms but not the effect of injunctive norms.

The effect of outcome expectancy on intention is solid and consistent and aligns with predominant theories on the role of behaviour outcomes as a motivator (Bandura, 1997; Fishbein & Ajzen, 1975). A robust result of the present study is that descriptive norms can influence the perception of outcome expectancy. Our results extend Moussaoui and Desrichard’s (2017) investigation of the link between descriptive norm and outcome expectancy on pro-environmental actions by adding health behaviours. The inclusion of injunctive norms, as well as competitor mediators (normative and informational social influence), allows a broader understanding of the mechanism underlying conformity to the norm, although some results are in unexpected directions.

Contrary to expectations, outcome expectancy was also influenced by injunctive norms in Study 2. This result would suggest that not only knowing what others do influences the perception of impact but also what is approved. A possible explanation is that the difference between injunctive and descriptive norms is not as clear in people’s minds. Eriksson et al. (2015) showed that both types of norms could be mixed up when participants are asked to recall social norms. Because this effect occurred only in Study 2, the post-COVID pandemic context can have accentuated this effect. For example, it can be imagined that on the topic of hand hygiene, others’ approval and action were assumed high, given heightened health risks. Descriptive and injunctive norms might have become intertwined because, during a global pandemic, individuals might assume people will act on what they approve, thus blurring the difference between the norms. This explanation is speculative, and more data is needed on this question. Future studies should further explore the distinction between norm types and the implication of communicating one on the perception of the other’s level. There are potentially significant implications for behaviour change techniques if future studies replicate the effect of injunctive norms on outcome expectancy and rule out that it is only due to a blurred distinction between both in the context of the pandemic. In the cases where descriptive norm could not be used without deception to increase outcome expectancy because it is a minority position or too high (which could create free-riding), injunctive norm could be communicated if its level is adequate.

Researchers have discussed that norm manipulations could only work if it is not too far from what people already think to remain believable (Croson & Shang, 2013; Reingen, 1982, Study 5). Studies design led to one experimental condition where participants learned about a low injunctive norm and a high descriptive norm about hand hygiene behaviour. This combination might not have been believable, and, indeed, in this condition, there was a reduction in reported informational social influence in Study 1 and no difference with the low descriptive/low injunctive condition in Study 2. This pattern suggests that the low credibility of the norm pattern (low injunctive/high descriptive) might counteract the positive effect of descriptive norms compared to a low injunctive/low descriptive condition. Future research could examine more targeted behaviours whereby an action may be considered behaviourally normative in people performing it without the accompanying social approval. Behaviours where there are social or financial costs for non-performance but often a lack of shared goodwill concerning performance (e.g., paid parking in public spaces, mandatory mask wearing) may fit the requirements of people performing the behaviour but not generally approving of it. Comprehensive piloting of behaviours with credible combinations of injunctive/descriptive norm may be beneficial, with message credibility checks (e.g., Mollen et al., 2023) potentially assisting in the interpretation of findings. In addition, Wang and Brown-Devlin (2022) showed that norm-based messages interacted with one’s own normative beliefs. In our study, we did not measure the initial normative beliefs of participants but future studies could test how consistency of the message with initial beliefs affects outcome expectancy.

A limitation of both studies is the use of items created for the purposes of the present research (e.g., normative and informational social influence) in the absence of established scales. Given our reliability indicators were satisfactory, it provides confidence our results are reliable although replication using the same items would be beneficial.

Another limitation is the inability to rule out alternative models, such as serial mediation instead of parallel mediation, because we measured all three mediators simultaneously. Longitudinal studies or experimental manipulation of the mediators would provide greater insights into the relationships among the variables we measured.

Regarding practical implications, our results assume even more importance in the post-pandemic context. They suggest that communication to promote hand hygiene should consider norm effects in conjunction with outcome expectancies. The social dilemma dimension of protective behaviours to prevent COVID-19 transmission has been highlighted (Johnson et al., 2020; Ling Hoh Teck & Chyong, 2020; Moussaoui et al., 2020). Social dilemmas might trigger beliefs of low outcome expectancy, also called drop-in-the-bucket (Attari et al., 2014). Norms to increase outcome expectancy is a new strategy that needs further testing in health social dilemmas. More broadly, many behaviours have a collective dimension and this characteristic should be considered when trying to understand their determinants. Our study shows that what others do and approve can influence the perception of outcome expectancy for those behaviours. Thus, when trying to promote collective behaviours such as protective behaviour in the context of a pandemic, but also pro-environmental behaviours, any mention of how many people are already doing the behaviour has an effect not only through social influence but also through the perception of one’s action impact. Care in what level of norm is communicated is, thus, required given the multiple mechanisms that underlie the influence of norms so as to avoid triggering a low expectancy perception.

Future studies could integrate a broader perspective on norms, including personal norms. In Thøgersen’s (2006) taxonomy, norms follow a continuum from external (descriptive norms) to internal (integrated norms), with injunctive/subjective social norms situated between these two continuum ends. In this study, our theoretical focus was on the mediators of the two most external types of norms in the taxonomy but those norms have been shown to be associated with personal norms in the context of collective behaviour (pro-environmental behaviours in the study by Thøgersen). A broader model would be worth testing in the future.

In conclusion, outcome expectancy is an important antecedent of intention in the case of collective pro-social behaviour. Preliminary evidence supports the notion it can be considered as an additional mechanism underlying the effects of norms alongside other established social influence constructs, such as normative and informational social influence. Future research should continue to examine the proposition that outcome expectancy is an important mechanism in conformity to the norm to extend our understanding of how descriptive and injunctive norms influence people’s intentions and behaviours.

## Data accessibility statement

Databases and syntaxes are available on OSF https://osf.io/ndf28/?view_only=3e40ae6efa834447b85c5dfe563656ad.

## Additional File

The additional file for this article can be found as follows:

10.5334/irsp.806.s1 Appendix. Power analysis and supplemental results (sensitivity analysis Study 1).

## Footnotes

https://osf.io/ndf28/?view_only=3e40ae6efa834447b85c5dfe563656ad.

Please note that we mention in the pre-registration the test of mediated moderation. However, this erroneously resulted from the previous studies (available on OSF) in which the experimental design was Type of norm (descriptive vs. Injunctive) X Level of norm (low vs. high), and for which the mediated moderation adequately tested our hypothesis. We realized that it is not the case for the two studies reported in this paper because we changed the experimental design. For this reason, the analysis deviate slightly from the pre-registration, as we no longer focus on the interaction between the types of norms, instead on the test of the mediation.

In the preregistration, the planned *N* is different because we used Schoemann et al. (2017) method for power calculation following a Reviewer’s comment.

The final sample size is, thus, lower than the expected sample size. As mentioned in the preregistration, we planned to conduct the analysis if at least 50% of the expected sample size was obtained, with the power issue kept in mind for interpretation.

In the preregistration, the planned *N* is different because we used Schoemann et al. (2017) method for power calculation following a Reviewer’s comment.

Study B reported online https://osf.io/ndf28/?view_only=3e40ae6efa834447b85c5dfe563656ad.
