# Supplementary material for: Collective Behaviours: Mediation Mechanisms Underlying the Influence of Descriptive and Injunctive Norms
Source: Int Rev Soc Psychol. 2023 Nov 13;36:15. doi: 10.5334/irsp.806 (PMC12372769; doi:10.5334/irsp.806)

Appendix

Power analysis Study 1

A priori power analysis

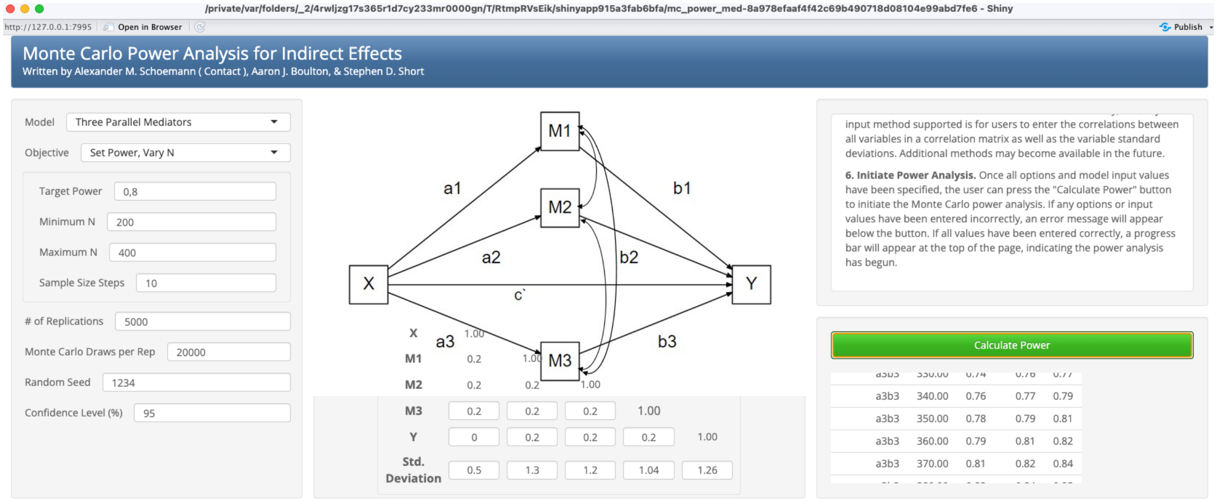

Sensitivity power analysis

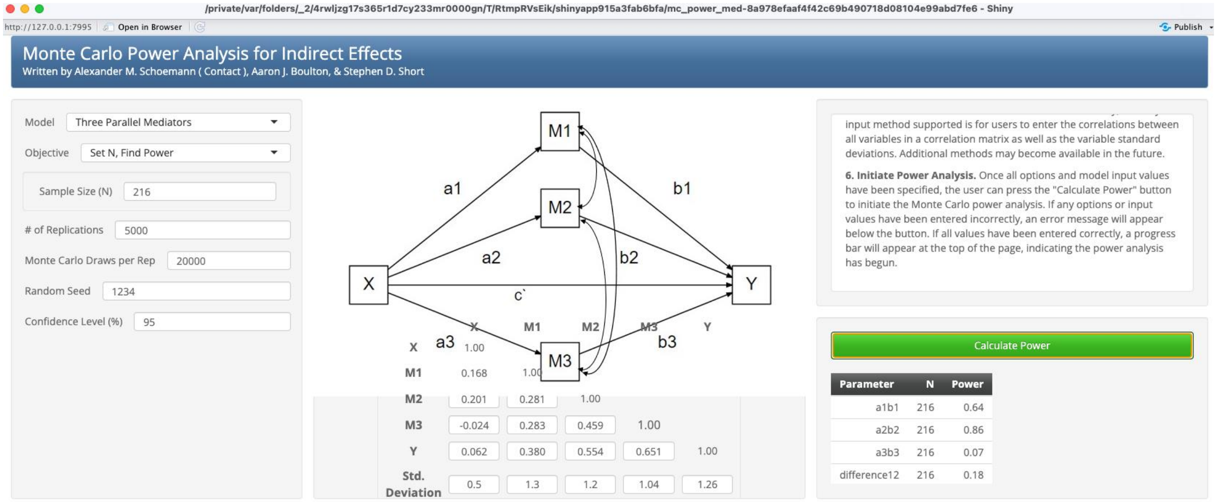

Sensitivity analysis Study 1: control for past behaviour

Component approach results

Path a_1. When controlling for past behaviour, the effect of descriptive norm on outcome expectancy (mediator 1) remained significant, $F(1,209) = 5.29$, $p = .022$, $\eta^2_p = .025$. The effect of injunctive norm level remained non-significant, although it became closer to

significance threshold, $F(1,209) = 2.97$, $p = .086$, $\eta^2_p = .014$. Past behaviour significantly affected outcome expectancy, $F(1,209) = 8.71$, $p = .004$, $\eta^2_p = .040$.

Path $\boldsymbol{a_2}$**.** Similarly, controlling for past behaviour did not change the results on normative social influence, as both types of norm effect on the second mediator remained significant: injunctive norm level, $F(1,209) = 18.10$, $p < .001$, $\eta^2_p = .080$; descriptive norm level, $F(1,209) = 4.85$, $p = .029$, $\eta^2_p = .023$. Past behaviour significantly affected normative social influence, $F(1,209) = 52.54$, $p < .001$, $\eta^2_p = .201$.

Path $\boldsymbol{a_3}$**.** Controlling for past behaviour, the effects of norms on informational social influence increased but did not reach significance threshold: injunctive norm level, $F(1,209) = 3.66$, $p = .057$, $\eta^2_p = .017$; descriptive norm level, $F(1,209) = 2.48$, $p = .117$, $\eta^2_p = .012$. The effect of past behaviour on informational social influence, $F(1,209) = 56.42$, $p < .001$, $\eta^2_p = .213$.

Path $\boldsymbol{b_1}$**.** The effect of M1 (outcome expectancy) on Y remained significant, $F(1,211) = 18.39$, $p < .001$, $\eta^2_p = .080$. Past behavior significantly affected intention, $F(1,211) = 91.95$, $p < .001$, $\eta^2_p = .304$.

Path $\boldsymbol{b_2}$**.** Similarly, the effect of M2 (normative social influence) on intention remained significant, $F(1,211) = 38.11$, $p < .001$, $\eta^2_p = .153$.

Path $\boldsymbol{b_3}$**.** The path from M3 (informational social influence) to intention also remained significant, $F(1,211) = 77.45$, $p < .001$, $\eta^2_p = .268$.

Path c. When controlling for past behaviour, the main effects of norms remained non-significant (descriptive norm: $F(1,209) = 0.03$, $p = .859$, $\eta^2_p = .000$; injunctive norm: $F(1,209) = 0.95$, $p = .330$, $\eta^2_p = .005$), and the interaction became non-significant, $F(1,209) = 1.84$, $p = .177$, $\eta^2_p = .009$. The effect of past behaviour on intention was significant: $F(1,209) = 101.03$, $p < .001$, $\eta^2_p = .326$.

Path c'. Controlling for past behaviour, mediators entered in the analysis retained their significant effect on intention (outcome expectancy: $F(1,206) = 9.65$, $p = .002$, $\eta^2_p = .045$; normative social influence: $F(1,206) = 17.98$, $p < .001$, $\eta^2_p = .080$; informational social influence, $F(1,206) = 47.30$, $p < .001$, $\eta^2_p = .187$). Main effects of norms and their interaction were non-significant (descriptive norm: $F(1,206) = 0.34$, $p = .558$, $\eta^2_p = .002$; injunctive norm: $F(1,206) = 1.89$, $p = .170$, $\eta^2_p = .009$; interaction: $F(1,206) = 0.04$, $p = .839$, $\eta^2_p = .000$). The effect of past behaviour on intention remained significant: $F(1,206) = 23.58$, $p < .001$, $\eta^2_p = .103$.

Index approach results

We predicted that the descriptive norm effect would be mediated by normative social influence, informational social influence, and outcome expectancy, while normative and informational social influences only (not outcome expectancy) would mediate the effect of injunctive norm on intention. A PROCESS macro was performed to analyse the parallel mediation. Coefficients are presented in Figure S1.

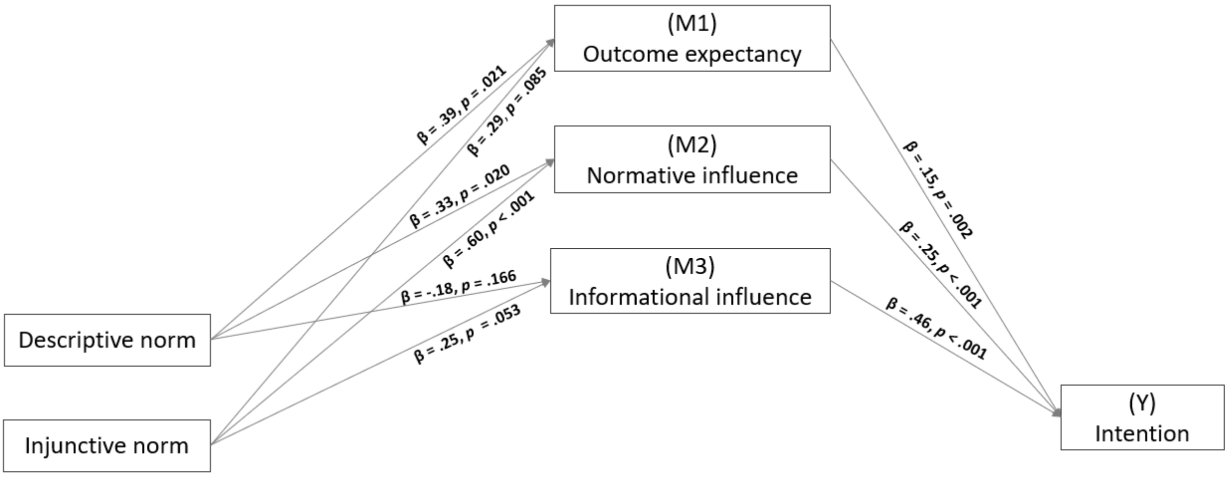

Figure S1. Parallel mediation model with past behaviour controlled for (Study 1)

Indirect effects show outcome expectancy mediated the link between descriptive norm level and intention, *b* = 0.06, 95% CI [0.004 to 0.124]. Normative social influence also mediated the link between descriptive norm level and intention, *b* = 0.08, 95% CI [0.01 to 0.17], while this was not the case for informational social influence, *b* = -0.08, 95% CI [-0.21 to 0.03]. The direct effect of descriptive norm on intention was non-significant when mediators were entered in the model, *b* = -.07, 95% CI [-0.30 to 0.17], *p* = .569.

Indirect effects show outcome expectancy did not mediate the link between injunctive norm level and intention, *b* = 0.04, 95% CI [-0.01 to 0.11], but that normative social influence and informational social influence both mediated the effect of injunctive norm level on intention, respectively *b* = 0.15, 95% CI [0.06 to 0.27], and *b* = 0.11, 95% CI [0.01 to 0.25]. The direct effect of injunctive norm on intention was non-significant when mediators were entered in the model, *b* = -.17, 95% CI [-0.40 to 0.07], *p* = .169.

Power analysis Study 2

A priori power analysis

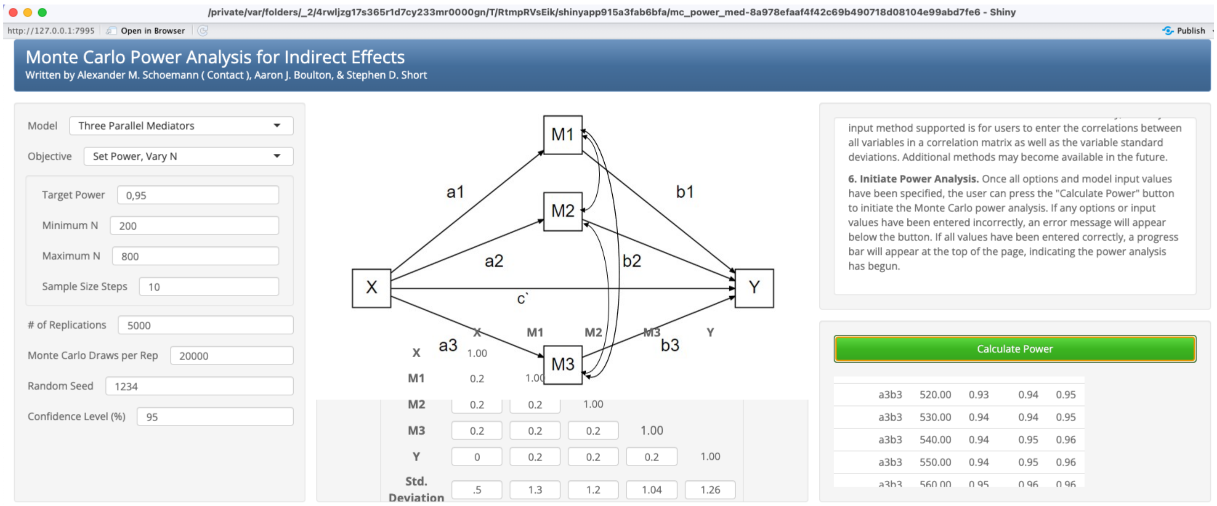

Sensitivity power analysis

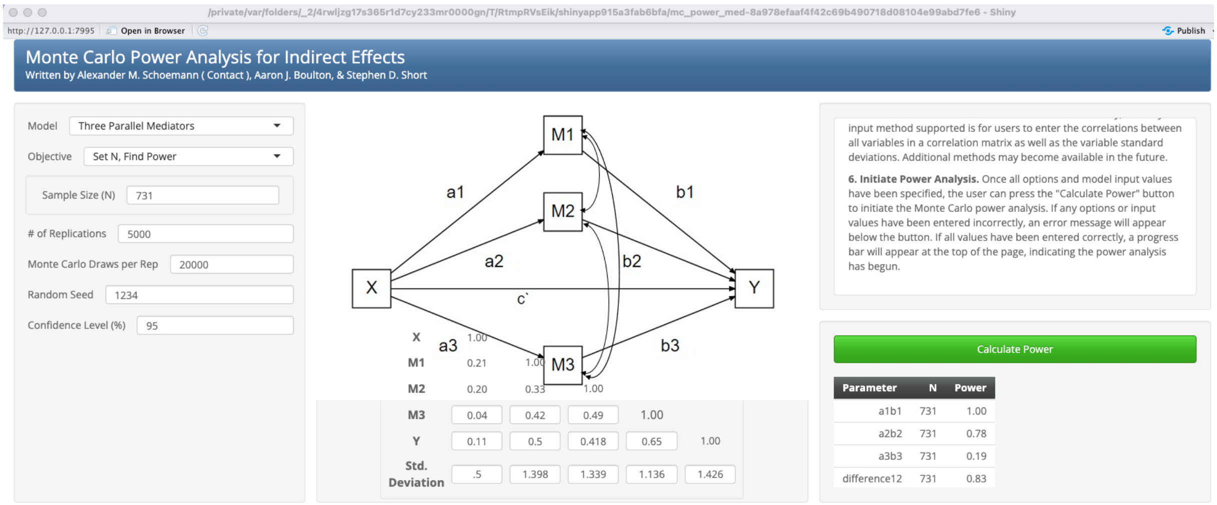

Supplement: Appendix. — Power analysis and supplemental results (sensitivity analysis Study 1). [file irsp-36-806-s1.pdf]
